# Experimental Verification of the Impact of the Contact Area between the Defect Site and the Scaffold on Bone Regeneration Efficacy

**DOI:** 10.3390/polym16030338

**Published:** 2024-01-26

**Authors:** You Min Kim, Min-Soo Ghim, Meiling Quan, Young Yul Kim, Young-Sam Cho

**Affiliations:** 1 Division of Mechanical Engineering, Wonkwang University, 460 Iksandae-ro, Iksan 54538, Republic of Korea; youmin@wku.ac.kr (Y.M.K.); msghim5834@naver.com (M.-S.G.); 2 Department of Pathophysiology, School of Basic Medical Sciences, Beihua University, Jilin 132021, China; mlquan225@beihua.edu.cn; 3 MECHABIO Group, Wonkwang University, 460 Iksandae-ro, Iksan 54538, Republic of Korea; 4 Department of Orthopedic Surgery, Daejeon St. Mary’s Hospital, Catholic University of Korea, 64 Daeheung-ro, Daejeon 34943, Republic of Korea

**Keywords:** scaffold, shape conformity, contact area, bone regeneration efficacy

## Abstract

In the field of bone tissue engineering, which is being developed for the ideal restoration of bone defects, researchers are exploring the improvement of the bone regeneration efficacy of scaffolds through various approaches involving osteoconductive, osteoinductive, and angiogenic factors. In the current trend of research, there is also a suggestion that the topological factors of recent scaffolds may influence the attachment, migration, proliferation, and differentiation of bone cells. Building upon experimental confirmation of the effect of scaffold conformity with the defect site on enhanced bone regeneration in previous studies, we conducted this research to experimentally investigate the relationship between contact area with the defect site and bone regeneration efficacy. The results demonstrated that as the contact area of the scaffold increased, not only did the resistance to bone tissue growth increase, more significant bone regeneration also occurred, as evidenced through histological analysis and micro-CT analysis. This research confirms that the contact area between the scaffold and the defect site is a critical variable affecting bone regeneration efficacy, emphasizing its importance when designing customized scaffolds. This finding holds promising implications for future studies and applications in the field.

## 1. Introduction

Additive manufacturing (AM) scaffolds refer to functional three-dimensional structures made from biocompatible materials, such as natural or synthetic polymers, and specific metal materials [1,2,3,4,5,6,7,8]. These scaffolds are developed in the field of bone tissue engineering to overcome limitations like immune rejection, infection transmission, and lack of donor site, with the aim of achieving ideal bone regeneration [9,10,11,12,13]. In AM scaffolds, materials with bone conductivity and osteoinductivity are frequently utilized to enhance the bone regeneration efficacy of the scaffold. These materials have been reported to recruit osteogenic cells around the defect or differentiate stem cells into bone cells [14,15,16,17,18]. Studies developing scaffolds with these materials have shown enhanced bone regeneration efficacy compared to the use of synthetic polymers alone [14,15,16,17,18]. Particularly, according to previous research, the highest bone regeneration efficiency was observed when a hydrogel containing osteoinductive and vascular inductive factors was radially incorporated into a scaffold made with a composite material containing bone conductive factors [18]. This suggests that not only the material composition but also the presence and pattern of scaffold pore are crucial factors in bone regeneration.

The presence and pattern of pore, as well as the shape conformity of scaffolds tailored in previous studies [19], have shown clues that they influence bone regeneration. In our previous study [19], we reported animal experimental results addressing bone regeneration related to a distinct “8-shape” calvarial defect. These assessments served as preliminary non-clinical evaluations tailored towards patient-specific bone regeneration in tissue engineering. Within these studies, the dimensions of the “8-shape” defect varied due to the creation of the shape from two circular defects, with the distance between their centers not being standardized. This led to variability in the defect’s dimensions. Therefore, to fabricate a scaffold compatible with this unique defect, we employed micro-CT scanning to obtain defect images. These images were subsequently converted to STL formats, facilitating their use in 3D printing (or AM) for fabricating an “8-shape” scaffold for bone regeneration. During these studies, an important observation was obtained. This was the non-negligible effect of conformity between the defect and the scaffold in the efficacy of bone regeneration.

Also, in a following study [20], we assessed a quantitative analysis of the effect of conformity (intimate contact between defect and scaffold). We found that this conformity is extremely important for achieving optimal bone regeneration. This focused investigation [20] demonstrated bone regeneration ability with respect to quantitatively controlled intimate contact area between the scaffold and the defect site. The results indicated the significance of conformity between scaffold and defect site in promoting new bone growth, its subsequent development, and maturation. Interestingly, despite our attempt to linearly increase contact areas in our experimental samples, remarkable bone regeneration was observed in scaffolds maintaining full intimate contact. This revealed that perfect conformity between the scaffold and defect site is crucial for maximizing bone regeneration efficacy.

Meanwhile, in our previous study, a particular design parameter issue was condoned. The control scaffold employed a rectangular grid-stacking configuration, whereas the experimental scaffold integrated both the rectangular grid-stacking and radial-stacking configuration. This combined design for the experimental scaffold was chosen to simplify the design for linearly increased contact area. This design variation did not compromise our important observation: the critical importance of perfect intimate conformity for effective bone regeneration. Nonetheless, based on our practical experience and indirect evidence of the effect of radial-patterned pores on bone regeneration [18], there is a convincing need to further investigate the influence of conformity between scaffold and defect site exclusively without the difference of pattern configuration.

**Figure 1:**
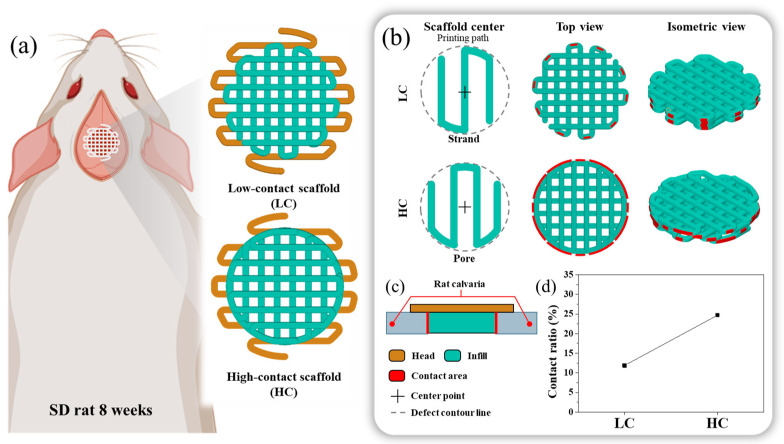
Schematics of the in vivo 3D printed scaffold: (**a**) Design images of scaffolds with low contact with the defect site and scaffolds with high contact with the defect site, (**b**) Differences in scaffold design approach and highlighting of the contact region between the designed scaffold and the defect site, Here, the low contact scaffold is designed with strands at the center, while the high contact scaffold is designed with pores at the center. (**c**) Cross-sectional view of the implanted scaffold, (**d**) Contact ratio between the designed scaffold and the defect site.

To do so, we used PCL to print two types of scaffolds with a porosity of about 50% and different contact areas with the defect, as shown in Figure 1, using a 3D printer with an applied extrusion method. These two types of scaffold have same rectangular grid-stacking configuration to eliminate the effect of the pattern’s difference. Afterward, we compared the variables in the designed and the measured variables after printing. To analyze the difference in bone regeneration ability according to the contact area between the scaffold and the defect, we implanted the scaffold into a rat calvarial defect, obtained micro-CT image data and tissue staining images, and performed image analysis. The new bone volume and histological maturity obtained from the image analysis results were then analyzed with respect to the contact area between the scaffold and the defect.

## 2. Materials and Methods

### 2.1. Materials

The biocompatible and biodegradable polymer PCL ($M_{W}$ = 37,000, Polysciences Inc., Warrington, PA, USA) served as the scaffold material. PCL pellets were introduced into an additive manufacturing barrel, where the temperature was elevated to 80 °C, and the PCL was melted over a period of 1 h. For the fabrication of scaffolds, a precision nozzle with an inner diameter of 400 µm was employed. The molten PCL was extruded through the nozzle using compressed air at a pressure of 500 kPa, with printing carried out at a rate of 0.3–0.5 mm/s to systematically build the scaffold and regulate its porosity. The creation path and design of the scaffold were executed using the 3D CAD program, SolidWorks (version 2020, Dassault Systems SolidWorks Corp., Waltham, MA, USA). The scaffold 3D printing path was G-coded based on the X-Y-Z path of the designed scaffold, taking into account the thickness of the strands extruded from the nozzle.

The weight of the scaffolds was assessed using a high-precision digital balance (HS204, Hansung, Seoul, Republic of Korea) with an accuracy of 0.2 mg, aiming to calculate the scaffold’s porosity. Following that, an optical microscope (Leica DMS1000, Leica Microsystems, Wetzlar, HE, Germany) was utilized to ascertain both the pore size and morphological characteristics of the scaffolds. The measurement of the contact ratio between the scaffold and the defect area was conducted by utilizing Image J (version 1.53q: National Institutes of Health, Bethesda, MD, USA), an image measurement tool, based on optical microscope images. 

### 2.2. Scaffold Design

In this study, we designed two types of scaffolds, as shown in Figure 1a, with the same channel orientation but varying contact areas between the defect and the scaffold. As both scaffold designs needed to be transplanted onto rat calvarial defects under identical conditions, their external dimensions were uniformly structured. The scaffold’s external configuration was designed with a diameter of 8 mm and a thickness of 1 mm. To ensure transplant stability and prevent contact with the brain when the scaffold was transplanted into the hole defect, as illustrated in Figure 1, the head was designed to be 2 mm larger than the defect size. The scaffold’s external dimensions were based on the naturally unhealable critical defect size, considering an 8 mm diameter hole defect as a reference for rat calvarial healing. The scaffold thickness was designed to be 1 mm to mimic the surrounding bone thickness, preventing the scaffold from touching the brain when the scaffold’s head was suspended in the surrounding bone tissue. Both scaffold designs were crafted with a porosity of approximately 50%, consistent with conditions reported in previous studies on additive manufacturing scaffolds [16,17,18,19,20].

For the contact area, which is an independent variable, one scaffold, with a relatively low contact area between the bone defect and the scaffold, was named the LC scaffold, and the other scaffold, with a relatively high contact area between the bone defect and the scaffold, was named the HC scaffold. To briefly explain the design process of each scaffold, as depicted in Figure 1b, the scaffold’s center and the presence of pores or strands influenced the number and length of contact points between the scaffold and the defect site. Therefore, we adopted this in the design, and the LC type was designed with a strand-centered design and the HC type was designed with a pore-centered design.

Here, considering the contact area and porosity of the scaffolds and aiming for similar pore size conditions, the LC scaffold and HC scaffold were designed with pore sizes of 500 μm and 590 μm, respectively. The range of scaffold pore sizes was selected within the effective range for bone regeneration, reported previously to be 100 to 1200 μm [21,22,23,24,25]. The difference in pore sizes between the two scaffolds was designed to be as similar as possible, taking into account previous research indicating no significant difference in bone regeneration efficacy within the range of 350 to 800 μm pore size difference [21].

### 2.3. Scaffold Characterization

**Figure 2:**
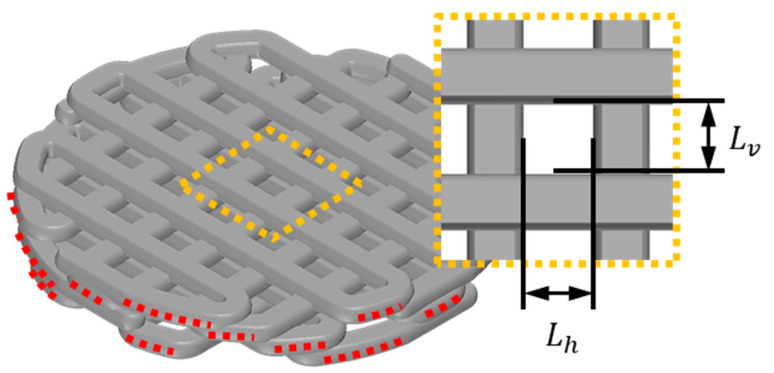
Illustration of essential variables for calculating scaffold contact area and pore size: the red dotted line symbolizes the contact strand length ($L_{C}$) between the scaffold and the defect, and the yellow dotted line offers an enlarged depiction of the scaffold’s pore. $L_{v}$ designates the vertical length of the pore, while $L_{h}$ signifies the horizontal length of the pore.

Generally, scaffold design commonly utilizes pore size and porosity as key variables for scaffold feature evaluation. In this study, the adjusted contact area was also measured, and all variables were compared to their design values. Firstly, the scaffold’s pore size was measured using an optical microscope, as described in the enlarged pore image in Figure 2. The horizontal length ($L_{h}$) and vertical length ($L_{v}$) of the pores were measured, and the average of these lengths was calculated as shown in Equation (1). The measurement of scaffold porosity was calculated using Equation (2). To briefly explain the porosity calculation, the weight of the fabricated scaffold ($m_{s}$) was measured. This weight was then divided by the known density of PCL ($\rho_{PCL}$ = 1.145 g/cm^3^) to calculate the volume of the fabricated scaffold. By subtracting the scaffold volume calculated from the scaffold’s outer volume ($V_{0}$), the volume of the pores was obtained. The ratio of the pore volume to the outer volume was then calculated to determine the scaffold’s porosity. Finally, the measurement method for the adjusted contact ratio in this study involved first using image analysis tools to measure the length of contacting strands between the defect area and the scaffold, denoted as contact length ($L_{C}$), as depicted by the red dashed lines in Figure 2. The sum of the measured contact lengths was then multiplied by the scaffold stacking thickness (*t*), which is 250 µm. This product was used to calculate the contact ratio. Since a direct comparison of contact area between the scaffold and the defect area is not intuitive, it was compared by calculating the ratio of the defect area ($A_{defect}$) to the contact area, as shown in Equation (3).

(1) Pore size (μm)=Lv+Lh2

(2) Porosity (%)=V0−msρPCLV0×100(%)

(3) Contact ratio (%)=(∑LC×t)Adefect×100(%)

### 2.4. Animal Preparation and Surgical Process

All animal experimentation was performed as authorized by the Institutional Review Board of St. Mary’s Hospital of Catholic University (CMCDJ-AP-2023-001). Sprague Dawley rats (eight-week-old, weight 240–260 g) were anesthetized via intraperitoneal injections of ketamine hydrochloride (Yuhan, Seoul, Republic of Korea) and Rompun^®^ (Bayer, Seoul, Republic of Korea). Surgical sites were shaved and sterilized with povidone, and then covered with a sterile drape. The rats were placed on a heated pad and covered with a sterile drape, except the tops of the skulls. To expose the calvarial bone, a longitudinal midline incision was performed, and the skin and periosteum were carefully separated. Using an 8 mm trephine bur, a circular defect (∅ = 8 mm) was created in the calvarial bone. The bone fragment was successfully removed and the defect was either left empty (n = 6) or implanted with the low-contact scaffolds (n = 6) and high-contact scaffolds (n = 6). The process was finished and closed with 4–0 black silk (Ailee, Seoul, Republic of Korea). All surgical procedures were performed under aseptic conditions, and the rats were kept on a heating pad during surgery and immediately postoperatively in order to maintain body temperature. To assist with post-operative pain management, each experimental animal was given an intraperitoneal injection of anti-inflammatory and analgesic drugs. To assess bone regeneration, rat calvarial samples were retrieved from the anesthetized rats in the fourth week after the implantation, and the harvested calvarial samples were fixed in 10% formalin.

### 2.5. New Bone Volume Analysis Based on CT Data

Micro-CT (1172, SkyScan, Kontich, AN, Belgium) measurements were taken at a resolution of 37 µm to analyze the bone regeneration ability of the contact area of the scaffold. All tomographic images were converted to DICOM files and the converted files were reconstructed into a 3D model using the MIMICS program (Materialise v 21.0, Leuven, VB, Belgium). In the reconstructed 3D model, the defect was masked and the new bone volume in the defect area was calculated using the program.

### 2.6. Histology

Hematoxylin and eosin (H&E) stain, Masson’s trichrome stain, and immunohistochemical (IHC) stain tests were used to study new bone histology and were performed after the harvested calvarial samples were subjected to micro CT scanning. The samples fixed with 10% formalin were again decalcified using ethylene-diamine-tetraacetic acid solution. After demineralization, the samples were embedded in paraffin and sliced into samples with 4 μm thicknesses. Afterward, the slides were stained with hematoxylin and eosin (H&E) and Masson’s trichrome stain. Masson’s trichrome stain was performed in accordance with the manual instruction of Masson’s trichrome stain kit (ab150686, Abcam, Cambridge, UK). The other slides were immunohistochemically stained for type I collagen (NB600-450, Novus Biologicals, Littleton, CO, USA) and osteocalcin (sc-365797, Santa Cruz Biotechnology, Santa Cruz, CA, USA) to evaluate new bone formation characteristics. In order to analyze the trends in collagen type I and osteocalcin protein staining from the IHC-stained images, the stained area relative to the total tissue area, excluding the scaffold area, was calculated using the Image J (Version 1.53q; National Institutes of Health, Bethesda, MD, USA) analysis tool.

### 2.7. Statistical Analysis

All data were expressed as mean and standard deviation values. Statistical analysis of new bone volume and histological quantitative analysis data was performed using single factor analysis of variance (ANOVA) using the SPSS program (Version 21.0; SPSS Inc., Chicago, IL, USA).

## 3. Results

### 3.1. Fabrication Fidelity

**Figure 3:**
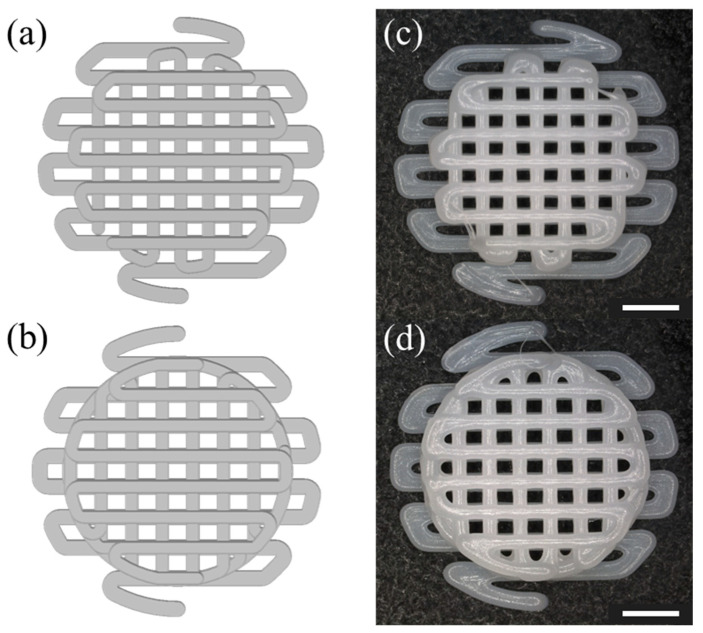
Image of the fabricated scaffold to be implanted into the defect: (**a**) top view of a HC scaffold designed in SolidWorks, (**b**) top view of a LC scaffold designed in SolidWorks, (**c**) optical image of a scaffold with HC (**d**) optical image of pore size on LC scaffolds. The scale bar is 2.0 mm.

The fabrication fidelity of the AM scaffolds was evaluated based on the defined parameters. To verify that the fabrication was successful in the actual fabrication, the design images were compared with the actual fabricated scaffolds. The PCL scaffolds were successfully fabricated as shown in Figure 3. The fabricated scaffold, as depicted in Figure 3c,d, was skillfully produced to closely resemble the design images in Figure 3a,b both morphologically and without any collapse or distortion in the porous regions.

**Figure 4:**
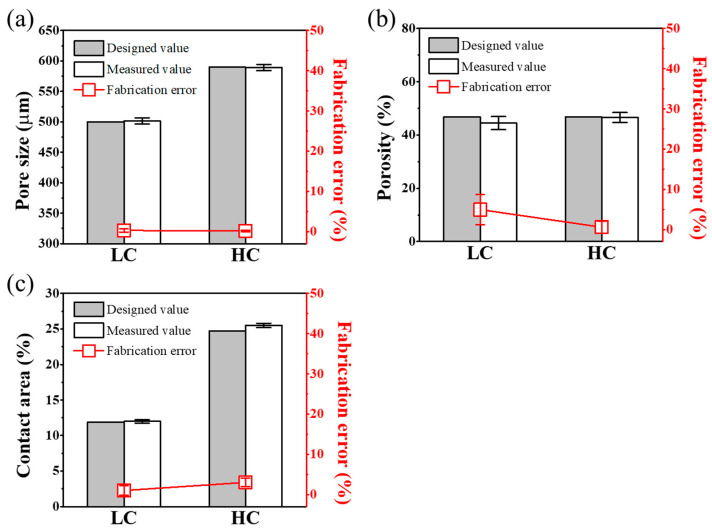
Scaffold’s parameters compared to designed and measured values: comparison graph of (**a**) pore size, (**b**) porosity, and (**c**) contact area of design and measured values of each scaffold.

We aimed to evaluate not only the morphological characteristics but also whether the scaffold design variables defined earlier, including pore size, porosity, and contact ratio, were accurately fabricated in a manner consistent with the design values. We aimed to assess the fidelity of fabrication by comparing the measured values of the defined parameters with the design values side by side, and the results are summarized in Figure 4. Detailed design values and measured values for each parameter are summarized in Table 1. As shown in Figure 4a, the designed values and fabrication error rates of the LC scaffold and HC scaffold, both designed with pore sizes of 500 μm and 590 μm, respectively, demonstrated a fabrication precision with an error rate of less than 1%. As depicted in Figure 4b, the porosity, with an error rate of less than 9%, was also confirmed to be precisely fabricated in accordance with the design. The measurement results of the contact ratio between the scaffold and the defect area, as shown in Figure 4c, confirmed that each scaffold was fabricated to be approximately twice the design value, closely resembling the design values.

### 3.2. Evaluate the Implantation Stability of a Scaffold

**Figure 5:**
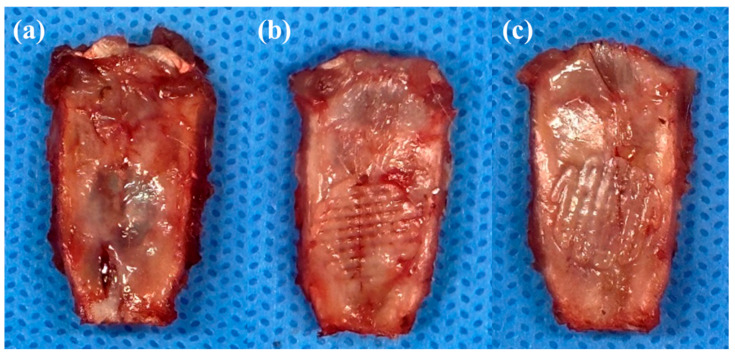
Images of each type of scaffold implanted into a rat calvarial defect: (**a**) rat calvarial defect. (**b**) LC scaffold implanted into a rat calvarial defect. (**c**) HC scaffold implanted into a rat calvarial defect.

These well-fabricated scaffolds were implanted into the rat calvarial defect, and the rats were sacrificed four weeks later to check the implantation status of the scaffolds. After four weeks, no delamination or hematoma was found in any of the rats, as shown in Figure 5. As shown in Figure 5b,c, each type of scaffold was well integrated with the surrounding tissue and covered with fibrous tissue.

### 3.3. Analyze In Vivo New Bone Formation

**Figure 6:**
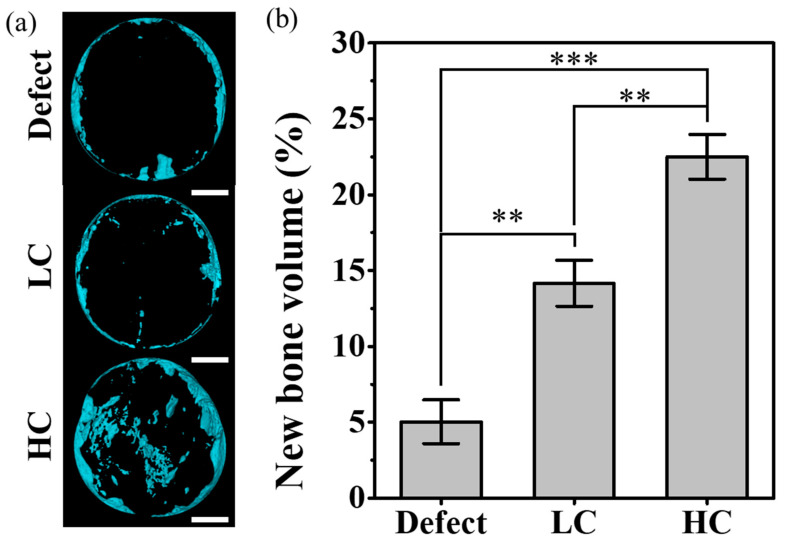
New bone volume for each type of scaffold implanted into a rat calvarial defect: (**a**) a reconstructive new bone image based on micro-CT data. The scale bar is 2.0 mm. (**b**) Graph of the ratio of the remaining pore volume to the volume of newly formed bone in the defect site (** *p* < 0.01, *** *p* < 0.001).

For quantitative analysis of the tissue images, the volume of the newly created 3D model was calculated. As shown in Figure 6, as the contact area of the scaffold increased, the volume of new bone also increased linearly. In addition, as shown in Figure 6a, the new bone in the case of defect was mainly generated at the edge close to the defect site, but the location of the newly generated bone moved closer to the center as the contact area increased. The graph shown in Figure 6b depicts the volumetric ratio of newly formed bone tissue relative to the volume of remaining pores in the entire defect. In the experimental group with only the defect, where the entire volume consisted of pores, a very small amount of newly formed bone, approximately 5%, was observed. On the other hand, in the experimental group where the LC scaffold was transplanted, the ratio of newly formed bone volume to the residual pore volume, excluding the proportion occupied by the scaffold, increased significantly to 14.16 ± 1.51%. Particularly in the case of the HC scaffold, when calculating the ratio of newly formed bone volume to the residual pore volume in the defect area, it exhibited the highest proportion of newly formed bone volume at around 22.49 ± 1.47%.

### 3.4. Histological Analysis

**Figure 7:**
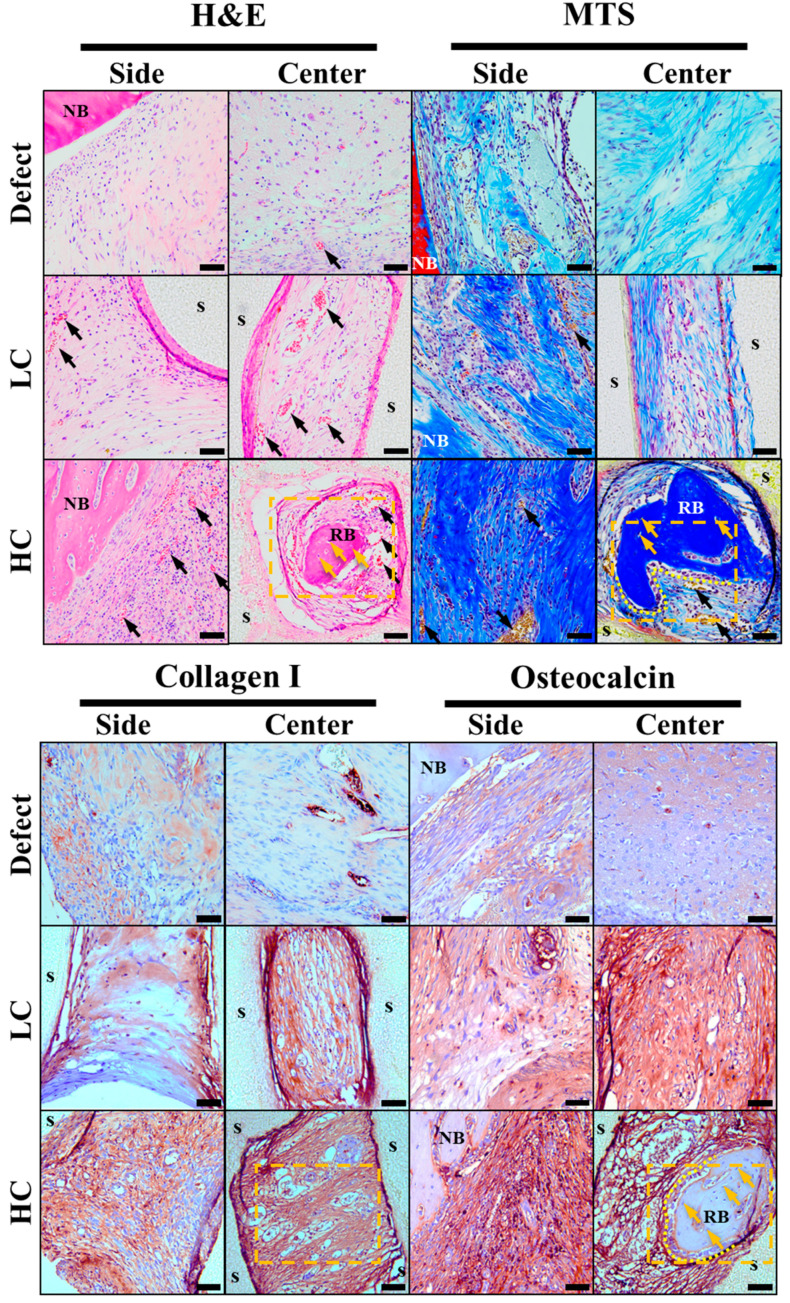
Histological staining results images. In the hematoxylin and eosin (H&E) stain images, cell nuclei, cytoplasm, and red blood cells are stained in purple, pink, and strong pink, respectively. In the Masson’s trichrome stain (MTS) images, cell nuclei, collagen, and red blood cells are stained in purple, blue, and yellow, respectively. In the immunohistochemistry (IHC) staining, collagen type I and osteocalcin (NB: native bone, s: scaffold, RB: regeneration bone, black arrow: new blood vessels, yellow arrow: new osteocyte, yellow dotted area: the location of the magnified image in the subsequent figure). The scale bar is 50.0 µm.

Four weeks after transplanting scaffolds created for critical defect size into rat calvaria, histological staining images were analyzed to examine bone regeneration behavior within the scaffold. For confirmation of regenerative behavior and scaffold ingrowth, observations were made at the side where the scaffold makes contact with the defect area and the central portion of the scaffold. These observations are summarized in Figure 7. Initially, when only the defect was present, regeneration behavior in the defect area was observed. In H&E staining results, the portion adjacent to native bone in the defect area exhibited a relatively higher density of cell nuclei and cytoplasm compared to the center of the defect. Additionally, vascularization evidence, indicated by rare congregations of red blood cells in the central region, was observed, as marked by black arrows in Figure 7. MTS results revealed a denser and bluer collagen staining around the native bone compared to the center of the defect, along with a relatively higher density of cell nuclei stained in purple. When bone defect occurred, collagen type I and osteocalcin were secreted throughout the bone tissue regeneration process, from cell proliferation to maturation, based on research reports, and collagen type I and osteocalcin were stained in brown accordingly [26]. The results revealed that the region proximal to the native bone displayed a significantly intensified staining for collagen type I and osteocalcin proteins compared to the central area. In summary, it was confirmed that bone regeneration in the region with only a defect occurs first on the lateral side.

Based on the staining results of the group with only a defect, the stained images of the groups with transplanted scaffolds were compared and observed. In the groups with transplanted scaffolds, the scaffold porous regions showed strong staining for cytoplasm, collagen, collagen type I, and osteocalcin protein in all staining results collectively. Particularly, in the HC scaffold, which is more closely adjacent to the defect area than other experimental groups, not only was the staining relatively strong compared to other groups, but more evidence of vascularization with densely packed red blood cells was also observed.

**Figure 8:**
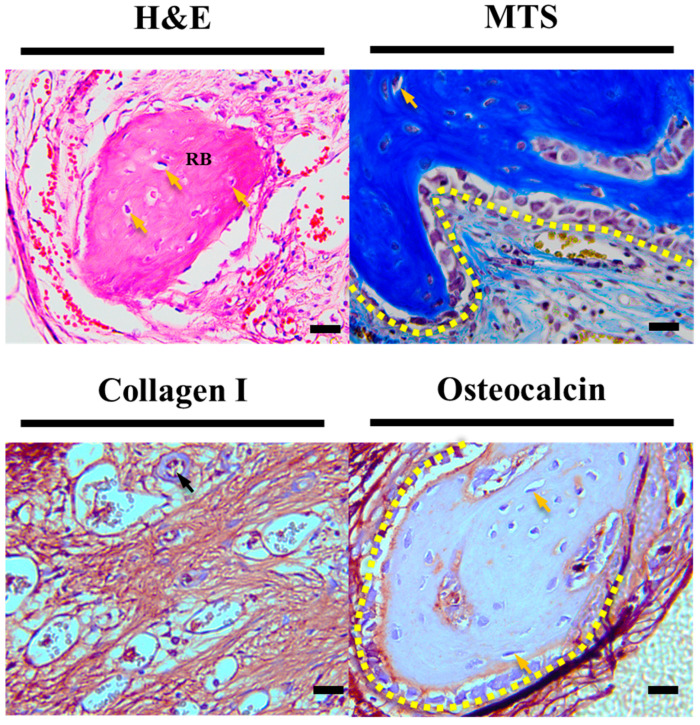
Enlarged histological staining images of HC scaffold center. The scale bar is 20.0 µm (RB: regeneration bone, black arrow: new blood vessels, yellow arrow: new osteocyte, yellow dotted area: lining osteoblast).

Furthermore, upon closer observation of the yellow dashed area in Figure 8, as magnified from Figure 7, in the HC scaffold group, not only was there more evidence of vascularization compared to other experimental groups, but also evidence of bone cell maturation during the bone regeneration process, such as osteocytes within the regenerated bone and surrounding lining osteoblasts, which were not observed in other experimental groups.

**Figure 9:**
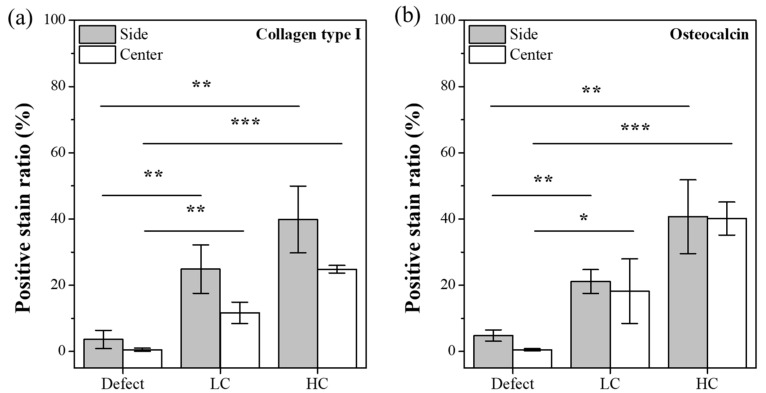
Positive stain ratio for IHC-stained images. (**a**) Trend graph for collagen type I. (**b**) Trend graph for osteocalcin protein (* *p* < 0.05, ** *p <* 0.01, *** *p* < 0.001).

Additionally, to investigate the differences in cell proliferation, maturation, and mineralization based on the contact area, we calculated the positive stain ratio for collagen type I and osteocalcin proteins through trend analysis of histological results. As depicted in Figure 9a, the staining for collagen type I, a protein known to be secreted throughout the proliferation, differentiation, and maturation processes of cells, showed significantly lower proportions in the defect group, particularly in the lateral and central aspects of the defect. In contrast, the groups with transplanted scaffolds exhibited relatively higher proportions of collagen type I secretion. Notably, it was evident that HC scaffolds had a higher ratio of collagen type I secretion in both the lateral and central regions compared to LC scaffolds. This trend was similarly observed for osteocalcin protein, as illustrated in Figure 9b. However, for osteocalcin, a protein secreted before mineralization, a similar ratio of secretion was observed in both lateral and central aspects of the groups with transplanted scaffolds, and this ratio demonstrated an increasing trend with the growing contact area.

## 4. Discussion

In this study, we aimed to experimentally verify the relationship between the contact area between the scaffold and the defect site and bone regeneration efficacy. Therefore, starting from scaffold design, we kept only the contact area as an independent variable while fixing other key design variables such as porosity, pore size, and channel direction. The porosity, pore size, and channel direction were designed based on dimensions reported in experiments using conventional additive manufacturing-based scaffolds [16,17,18,19,20]. Designing a scaffold structure with perfectly identical pore sizes while intentionally adjusting the contact area, while maintaining the same porosity, was challenging. Therefore, in this study, we had to devise a scaffold design method with identical porosity, referring to previous research reports indicating no significant difference in bone regeneration efficacy within pore sizes ranging from 350 to 800 μm [21]. Consequently, intentionally designing the LC scaffold with a pore-centric focus and the HC scaffold with a strand-centric focus aimed for an intentional difference of about 90 μm in pore size to match their porosity. This is because the HC scaffold, designed with strands at the center, has more strands internally within the same external size compared to the LC scaffold designed with a pore at the center. As a result, the HC scaffold, while having more contact with the defect site, paradoxically ends up having a lower porosity due to the higher number of strands inside within the same external size. Furthermore, through stained images in Figure 7, we experimentally reproduced and validated the reported finding that vascularization occurs in scaffolds with pore sizes of 300–500 μm and a porosity of 50%, observing more evidence of vascularization in all scaffolds compared to the experimental group with only defects [21,22,23,24].

Upon comparing the fabrication errors after producing the designed scaffold through the AM system, it was confirmed that the fabrication errors for all key variables were less than 9%, indicating precise fabrication. Given the close resemblance between experimental design and fabrication, it was concluded that experimental errors were more likely influenced by the design rather than fabrication errors. Therefore, scaffolds that were faithfully produced according to the design were deemed suitable for use in the experiments.

After implanting the precisely fabricated scaffold in a rat calvarial defect model for 4 weeks, the analysis of the differences in bone regeneration efficacy revealed a clear influence of the scaffold-to-defect contact ratio on bone regeneration. As shown in Figure 6, it demonstrated that bone ingrowth occurred in proportion to the contact area, with the quantity increasing from 14.16 ± 1.51% to 22.49 ± 1.47%, approximately 1.5 times higher. However, when comparing the contact area, it was observed that the relationship was not strictly proportional, as the contact area increased more than twofold, from 12.04 ± 0.85% for the LC scaffold to 25.63 ± 1.77% for the HC scaffold. Nevertheless, despite this non-proportional relationship, histological analysis results from stained images, as shown in Figure 7, Figure 8 and Figure 9, demonstrated that the contact area between the scaffold and the defect site reached a rapid bone maturation process during the same period. In the side portion of Figure 7, HC scaffold exhibited the highest cell nucleus density. As the contact area increased, this suggests a smoother influx of cells into the scaffold and subsequent proliferation. In particular, as seen in Figure 8, only the HC scaffold showed the presence of regenerated bone, osteocytes, and lining osteoblasts, indicating a significantly faster bone maturation process compared to other experimental groups. This aligns with previous studies reporting bone maturation and mineralization within approximately 4 weeks after fracture [27]. These trends clearly demonstrated that the contact area, as shown in Figure 9, not only influenced the bone maturation process and mineralization but also had an impact on ingrowth. Through these results, it was experimentally proven that the contact ratio between the scaffold and the defect site is a decisive factor influencing bone regeneration efficacy.

However, it is necessary to consider the relationship between the scaffold-to-defect contact ratio and the porosity. Designing the scaffold by considering only the contact area may result in a scaffold without side pore having the highest contact area. However, previous studies have reported that scaffolds with side pore show higher bone regeneration efficacy compared to scaffolds without side pore [28,29]. Therefore, for efficient bone regeneration, the relationship between the porosity and contact ratio of the scaffold needs to be appropriately considered. Considering the results of this study and previous research collectively, it can be inferred that at least the contact ratio between the scaffold and the bone defect, as well as the porosity of the scaffold, should be designed to be similar.

## 5. Conclusions

In summary, considering the overlooked pathway in previous research regarding the relationship between the defect–scaffold contact area and bone regeneration efficacy [20], this study aimed to experimentally verify the differences in bone regeneration efficacy when only the contact area was adjusted under the same channel direction conditions. First, to independently control the contact area, the scaffold was designed with a separation of the center into strands and pores, confirming the ability for significant contact area adjustment. In this process, the impact of pore size, generated to maintain porosity, was confirmed to be negligible based on reported studies [21]. Results from the transplantation of precisely designed scaffolds into rat calvaria with a critical defect size for 4 weeks revealed that HC scaffolds with a relatively higher contact area exhibited more bone regeneration compared to LC scaffolds and the defect group during the same period. Particularly, in the defect group or LC scaffold, bone formation was observed around the defect site, while in the HC scaffold with a relatively higher contact area, significant ingrowth occurred, leading to more pronounced bone regeneration. Experimental evidence, as shown in histological stain analysis, supported the idea that as the contact area increased, cells around the defect site exhibited higher cell density, and more cells migrated into the scaffold center, supporting increased proliferation. Furthermore, histological analysis results indicated signs of mature processes, such as regenerated bone, osteocytes, and osteoblasts lining, only in HC scaffolds with a relatively higher scaffold–defect contact ratio. Additionally, higher secretion of collagen type I and osteocalcin proteins in HC scaffolds compared to other experimental groups suggested faster progression toward bone maturation and mineralization. These experimental findings ultimately proved that the scaffold–defect contact area is a crucial and fundamental factor influencing bone regeneration efficacy. This emphasizes the importance of considering both the external form of regeneration and the shape of the defect in the design of customized scaffolds.

## Figures and Tables

**Table 1:** Definition of scaffold design parameters.

	Pore Size [µm]	Porosity [%]	Contact Area [%]
Low contact scaffold			
Designed value	500.00	46.77	11.87
Measured value	501.45 ± 2.48	44.41 ± 2.46	12.04 ± 0.85
High contact scaffold			
Designed value	590.00	46.81	24.73
Measured value	589.11 ± 2.35	46.49 ± 1.90	25.63 ± 1.77

## Data Availability

Data are contained within the article.
